# A comparative study on the three calculation methods for reproduction numbers of COVID-19

**DOI:** 10.3389/fmed.2022.1079842

**Published:** 2023-01-05

**Authors:** Buasiyamu Abudunaibi, Weikang Liu, Zhinan Guo, Zeyu Zhao, Jia Rui, Wentao Song, Yao Wang, Qiuping Chen, Roger Frutos, Chenghao Su, Tianmu Chen

**Affiliations:** ^1^State Key Laboratory of Molecular Vaccinology and Molecular Diagnostics, School of Public Health, Xiamen University, Xiamen, Fujian, China; ^2^Xiamen Center for Disease Control and Prevention, Xiamen, Fujian, China; ^3^Cirad, UMR 17, Intertryp, Université de Montpellier, Montpellier, France; ^4^Zhongshan Hospital, Fudan University (Xiamen Branch), Xiamen, Fujian, China

**Keywords:** COVID-19, reproduction number (R), definition methods, next generation matrix, serial interval *(SI)*

## Abstract

**Objective:**

This study uses four COVID-19 outbreaks as examples to calculate and compare merits and demerits, as well as applicational scenarios, of three methods for calculating reproduction numbers.

**Method:**

The epidemiological characteristics of the COVID-19 outbreaks are described. Through the definition method, the next-generation matrix-based method, and the epidemic curve and serial interval (*SI*)-based method, corresponding reproduction numbers were obtained and compared.

**Results:**

Reproduction numbers (*R*_*eff*_), obtained by the definition method of the four regions, are 1.20, 1.14, 1.66, and 1.12. Through the next generation matrix method, in region H *R*_*eff*_ = 4.30, 0.44; region P *R*_*eff*_ = 6.5, 1.39, 0; region X *R*_*eff*_ = 6.82, 1.39, 0; and region Z *R*_*eff*_ = 2.99, 0.65. Time-varying reproduction numbers (*R*_*t*_), which are attained by *SI* of onset dates, are decreasing with time. Region H reached its highest *R*_*t*_ = 2.8 on July 29 and decreased to *R*_*t*_ < 1 after August 4; region P reached its highest *R*_*t*_ = 5.8 on September 9 and dropped to *R*_*t*_ < 1 by September 14; region X had a fluctuation in the *R*_*t*_ and *R*_*t*_ < 1 after September 22; *R*_*t*_ in region Z reached a maximum of 1.8 on September 15 and decreased continuously to *R*_*t*_ < 1 on September 19.

**Conclusion:**

The reproduction number obtained by the definition method is optimal in the early stage of epidemics with a small number of cases that have clear transmission chains to predict the trend of epidemics accurately. The effective reproduction number *R*_*eff*_, calculated by the next generation matrix, could assess the scale of the epidemic and be used to evaluate the effectiveness of prevention and control measures used in epidemics with a large number of cases. Time-varying reproduction number *R*_*t*_, obtained *via* epidemic curve and *SI*, can give a clear picture of the change in transmissibility over time, but the conditions of use are more rigorous, requiring a greater sample size and clear transmission chains to perform the calculation. The rational use of the three methods for reproduction numbers plays a role in the further study of the transmissibility of COVID-19.

## Introduction

Ever since the first confirmed case of COVID-19 was reported in December 2019, there have been more than 500 million infections around the world, with a total of over 6 million deaths (1, 2). This pandemic has been a great challenge for not only people's health and the global health-care system but also for the socio-economy. In the last 2 years, multiple mutant variants of SARS-CoV-2 have emerged, which affects the transmissibility and severity of the virus greatly (3, 4). In China, due to the implementation of non-pharmaceutical (NPIs, such as quarantine, nucleic-acid testing, and social distancing) and pharmaceutical interventions (PIs, such as medication as well as vaccination) during the prevention and control of COVID-19, there were some satisfying results (1, 5–7). At the current stage of the pandemic, applying the appropriate quantitative index to describe the transmission dynamics of the disease and evaluating its transmissibility to propose corresponding controlling strategies have been areas of great interest for researchers.

In general, researchers use the secondary attack rate (SAR), the number of susceptible contacts who develop the disease as a percentage of the total number of susceptible contacts between the minimum incubation period and the maximum incubation period of certain infectious diseases, or reproduction numbers (R), the number of cases of second-generation infection caused by an infected individual with an infectious disease in a fully susceptible population without any intervention, to illustrate the transmissibility of the disease. As the modeling studies for infectious diseases are more sophisticated, the application of reproduction numbers has become more compelling, among which, basic reproduction number (*R*_0_), effective reproduction number (*R*_*eff*_), and time-varying reproduction number (*R*_*t*_) are commonly used (8). At the beginning of 2020, researchers calculated *R*_0_ of COVID-19 in Wuhan, China with the definition method, to demonstrate the transmissibility of this emerging infectious disease (EID) for the first time (9). Recently, there have been some studies published about obtaining *R*_*eff*_ and *R*_*t*_ by applying the next-generation matrix and serial interval (*SI*) (4, 10, 11). Simultaneously, there are studies about *R*_*eff*_ of COVID-19 by establishing transmission dynamics models, such as SEIAR models (1, 12, 13). Now that reproduction numbers are playing an important role in predicting and preventing infectious diseases, especially in COVID-19, it is of significance that we use the optimum reproduction number in an outbreak scenario with appropriate calculation methods.

In most cases, the reproduction numbers are obtained from definition methods, next-generation matrices, or using *SI* and epidemic curves (14–17). However, there are not enough studies about the optimum scenarios for the above calculation methods or they are misused in some circumstances. Therefore, in this study, we used data from four outbreaks occurring in China during 2021–2022, which were denoted as regions H, P, X, and Z, for assessing the transmissibility of COVID-19, and used reproduction numbers, which are obtained by the definition method, the next-generation matrix-based method, and *SI* and epidemic curve-based method, to be the indicators for COVID-19 transmissibility (1, 18–20). The epidemiological significance and application considerations of the reproduction numbers calculated using these three ways were also compared to provide a reference for public health departments to use more accurate reproduction numbers to formulate prevention and control measures and quantitatively evaluate the effects of various interventions.

## Methods

### Study design

**Figure 1:**
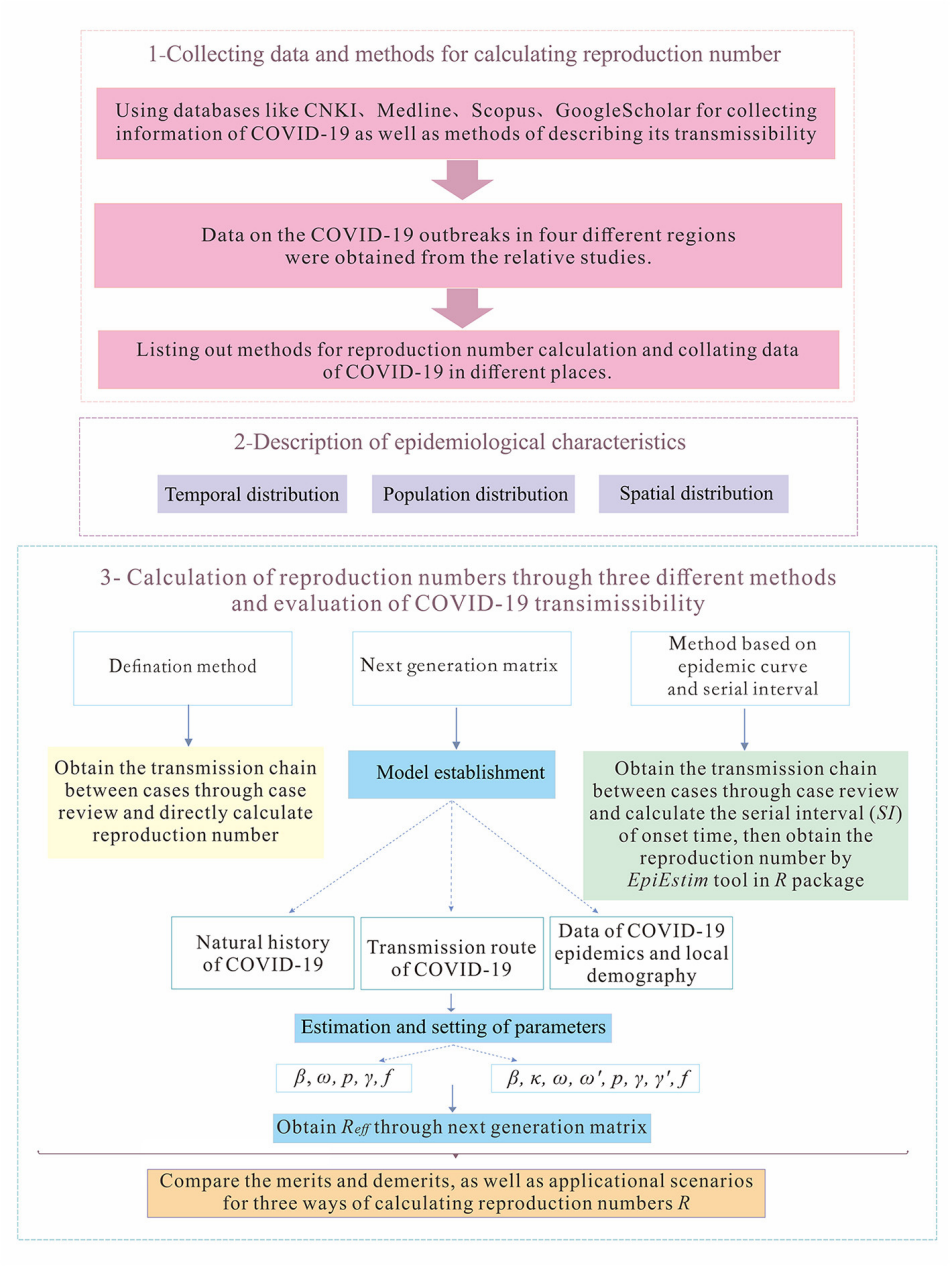
The framework of this study.

There are three major steps involved in this study (Figure 1). First, we collected data and methods for calculating reproduction numbers. Then, we described the epidemiological characteristics of the outbreaks in four regions, H, P, X, and Z. Finally, we obtained reproduction numbers of COVID-19 in those regions through three different methods, which are the definition method, the method based on the next-generation matrix, and method based on *SI* and epidemic curve, to evaluate transmissibility of the diseases under various circumstances.

### Data collection

In this study, data were collected on four COVID-19 outbreaks in China from corresponding studies and open accesses, which comprised of transmission caused by the Delta variant in region H (22 July−15 August 2021) (19), region P (4–23 September 2021) and region X (8–30 September 2021) (1, 20), and the Omicron variant in region Z (13–21 January 2022) (18). The data collected included basic information about the patients (age, sex, occupation, address, whether vaccinated, and doses), clinical typing (asymptomatic infection, minor, ordinary, heavy, and severe), key time points of transmission (date of exposure, date of onset, date of the positive test, and date of confirmation), and transmission chains between cases. Population information for each site was obtained from the 7th National Census data in 2020.

### Calculation of COVID-19 reproduction number

#### The definition method

Reproduction number, *R*, is defined as the average number of infections acquired by an infected person during an infected period in a susceptible population. Thus, the definition method means that the transmission chain of an outbreak is obtained and directly calculates the reproduction number, which is denoted as the basic reproduction number, *R*_0_. Specifically, first, we collect the number of second-generation cases caused by the first-generation cases and divide the number of second-generation cases by the number of first-generation cases to calculate the *R*_0_.


(1) $$\begin{array}{l} R_{0}=\frac{Sum\;of\;the\;number\;of\;second-generation\;cases\;transmitted\;per\;first-generation\;case}{Sum\;of\;the\;number\;of\;cases\;in\;first\;generation} \end{array}$$

### The next-generation matrix-based method

#### Model development

According to the natural history of COVID-19 and the epidemiological characteristics of the four regions, we grouped the total population N into Susceptible, S; Exposed, E; Infected, I; Asymptomatic, A; and Recovered/Removed, R. Thereby, a SEIAR (Susceptible—Exposed—Infected—Asymptomatic—Recovered/ Removed) transmission dynamics model was constructed.

The SEIAR model was built under these assumptions:

1) We set the infection coefficient after effective contact between a susceptible person S and an infection with symptoms I as *β*, also assuming that the transmissibility of an asymptomatic infection A is *κ* times that of a symptomatic infection I (0 < *κ* < 1), then the number of new infections at time t is *β*S(I + *κ*A).
2) At time t, there would be two results for exposed population E, they either become symptomatically infected I or asymptomatically infected A. Assume that a proportion p of E is converted to A, and the proportion of E to I is (1–*p*). It is generally understood that after a person is exposed to pathogens, there is a time interval between when he/she becomes invaded by the pathogen and when it is emitted, known as the latent period. The rate of transformation from E to A is proportional to the amount of E with a scale factor of *pω*′E, and *ω*′ is the latent period coefficient.
3) At time t, the number of transfers to the recovering population R is *γ*I if the time interval between onset and diagnosis from a symptomatic infection I is *γ*; the number of transfers to R from A, identified as an asymptomatic infection, is *γ*′I.
4) Since there is a possibility of death in symptomatic infections, the mortality rate is taken as *f* .

The functions of the SEIAR transmission dynamics model are as follows:


(2) $$\{\begin{array}{c} \frac{dS}{dt}=-\beta S\left(I+\kappa A\right)/N\\ \frac{dE}{dt}=-\beta S\left(I+\kappa A\right)/N-p\omega^{\prime }E-\left(1-p\right)\omega E\;\\ \;\frac{dI}{dt}=p\omega^{\prime }E-\gamma I-f\;I\;\\ \frac{dA}{dt}=\left(1-p\right)\omega E-\gamma^{\prime }A\\ \frac{dR}{dt}=\gamma I+\gamma^{\prime }A\\ N=S+E+I+A+R \end{array}$$

#### Parameter estimation

In this SEIAR model, there are various of parameters need to be estimated before modeling. The infection coefficient *β* is obtained by fitting the actual data in the model. The incubation period of the Delta variant is 3–7 days (4, 21) and that of the Omicron variant is 2 days (18, 22); therefore, we set the incubation period coefficient for the Delta variant as 1/*ω* = 0.33 and the Omicron variant as 1/*ω* = 0.4. As there were not any asymptomatic infections during the outbreaks in regions P, X, and Z, we set the proportion of asymptomatic infections as *p* = 0. While for region H, where asymptomatic infections were reported, we set its proportion of asymptomatic infections as *p* = 0.15 (23, 24), and we set the latent period coefficient in region H as 1/*ω*′ = 0.2. Simultaneously, according to the previous studies which illustrate that the infection coefficient of asymptomatic infections compared to symptomatic infections is 0.7 (21), we set *κ* = 0.7. It is widely accepted that the disease duration of the Delta variant is ~5 days, while it is 4–5 days for the Omicron variant (22), so we set the recovery rate of symptomatic infections as 1/*γ* = 0.2 and 1/*γ* = 0.22, respectively. As for the recovery rate for asymptomatic infections, we set 1/*γ*′ = 0.1 (24, 25). No region reported a fatal case, so the fatality of the disease is *f* = 0 (Table 1).

**Table 1:** The definition and values of parameters in the SEIAR model of COVID-19.

**Parameter**	**Definition**	**Unit**	**Value**	**Source**
β	Infection coefficient	1		Curve fitting
κ	Infection coefficient of asymptomatic infections compared to symptomatic infections	1	0^b^^,^ ^c^^,^ ^d^, 0.7^a^	(18)
ω	Incubation coefficient in symptomatic infections	Day^−1^	3^a, b, c^, 2.5^d^	(9, 17, 20)
*ω′*	Incubation coefficient in asymptomatic infections	Day^−1^	0^b^^,^ ^c^^,^ ^d^, 5^a^	(9, 18)
*p*	Proportion of asymptomatic infections	1	0^b^^,^ ^c^^,^ ^d^, 0.15^a^	Actual data
γ	Recovery or removal rate for symptomatic infections	Day^−1^	5^a, b, c^, 4.5^d^	(20)
*γ′*	Recovery or removal rate for asymptomatic infections	Day^−1^	10	(19)
*f*	Fatality of the disease	1	0	Actual data

a Is for region H,

b is for region P,

c is for region X,

d is for region Z.

This method is an indirect way to calculate the reproduction numbers that use a transmission dynamics model, denoted as effective reproduction number *R*_*eff*_. This refers to the expected number of second-generation cases that can be infected by the first-generation cases with certain effective interventions implemented (1, 11).

The formula for calculating the effective reproduction number *R*_*eff*_ is as follows: Refer to Supplementary material for detailed calculations (11).


(3) $$R_{eff}=\frac{\beta S/N}{p\omega^{\prime }+\left(1-p\right)\omega }\times \left[\frac{\kappa p\omega^{\prime }}{\gamma^{\prime }}+\frac{\left(1-p\right)\omega }{\gamma +f}\right]$$

#### *SI* and epidemic curve-based method

Through the transmission chains, which are obtained from the collected COVID-19 data, we count the standard deviations and means of serial intervals (*SI*) of onset dates of outbreaks. Then, we calculate the reproduction number, denoted as *R*_*t*_, by the *EpiEstim* package in *R* software. *R*_*t*_ represents the average number of second-generation infections from first-generation cases per unit time in a susceptible population. Refer to Supplementary material for a detailed calculation.


(4) $$\begin{array}{l} SI=onset\;time\;of\;second-generation\;cases\\ \;-onset\;time\;of\;first-generation\;cases \end{array}$$

### Statistical analysis

Data entry and organization related to this study were performed in *Excel 2019*. Continuous quantitative variables were described by median ± interquartile range (IQR) and categorical qualitative variables by percentages. Statistical analysis was performed using *SPSS* version 22.0, and differences were statistically significant at *p* ≤ 0.05. Graphs were plotted using *Graph Prism 7.0*; transmission chains were plotted using *OrignPro* version 2022; models were fitted using *Berkeley Madonna 8.3.18*; differential equations were solved using the fourth-order Runge Kutta method; and model convergence was based on the root least mean square (LRMS), further using the coefficient of determination (*R*^2^) to determine the goodness of fit. The R_t_ was calculated using the *EpiEstim* package in *R* software.

## Results

### Epidemiological characteristics of COVID-19 in the four regions

From 22 July to 15 August 2021, there were 129 COVID-19 cases caused by the Delta variant reported in region H, with the highest number of new cases in a single day being 15 on 1 August. In 2021, from 4 to 23 September, in region P, a total of 209 COVID-19 cases caused by the Delta variant were reported. On 10 September, the highest number of new cases in a single day, 42 cases, was reached. At the same time, there was another outbreak in region X from 8 to 30 September, which was an outflow of region P's outbreak. The COVID-19 outbreak in region Z in 2022 was a consequence of the Omicron variant, which had basically replaced the Delta variant and become the dominant variant throughout the world. This outbreak in region Z started on 13 January and lasted for 8 days, with a total of 38 cases.

**Figure 2:**
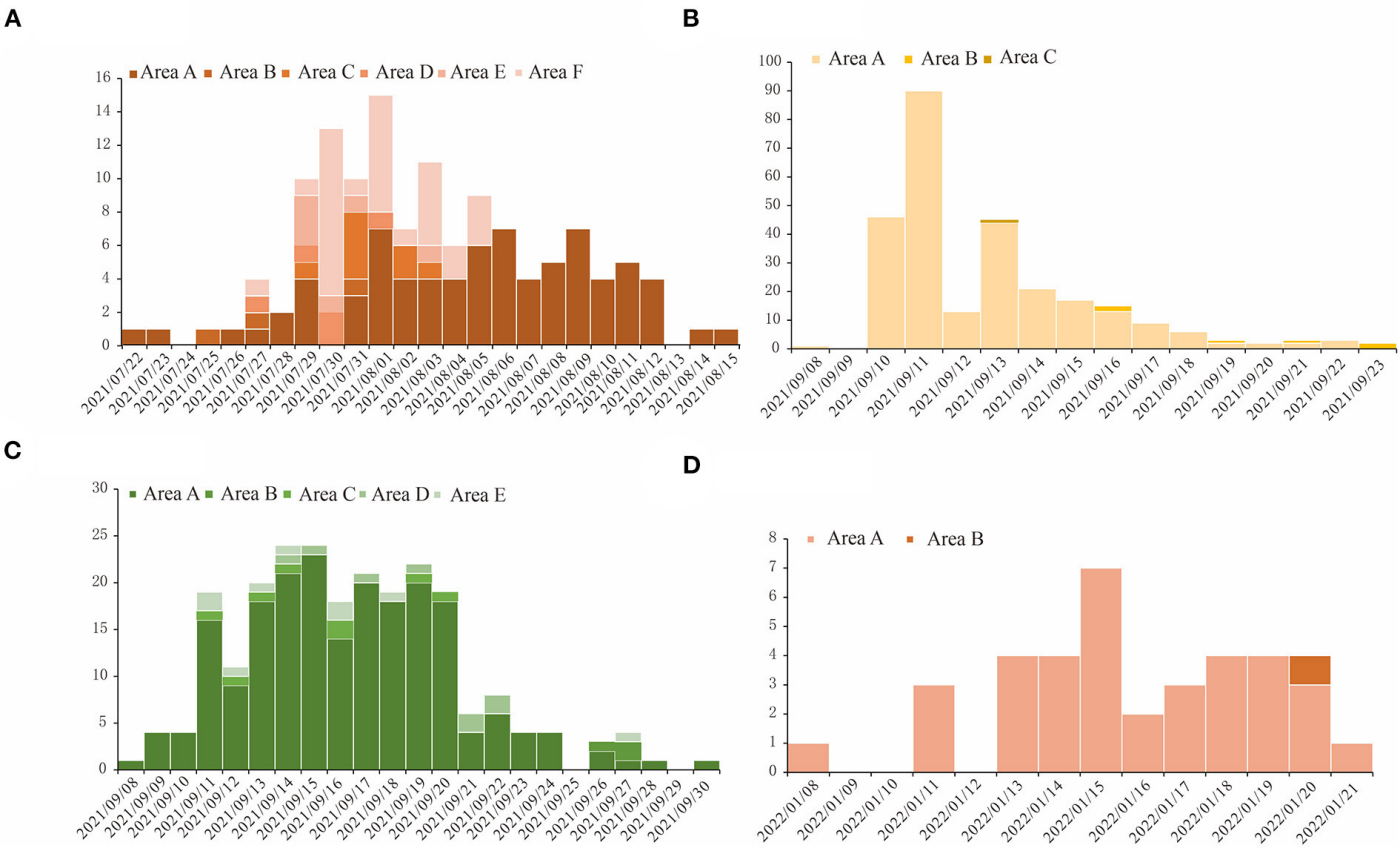
The temporal distribution for COVID-19 outbreaks in four regions studied in this research. The horizontal axis is the dates when the outbreaks happened, and we used different shades of color for the bars in each figure to show the number of cases in different areas in these regions. **(A)** Region H, **(B)** Region P, **(C)** Region X, and **(D)** Region Z.

The spatial distributions of cases in each of those four outbreaks give clues about how the outbreaks occurred. The outbreak in region H was scattered in seven districts in the region, with 58.9% of cases reported in area A of region H. During the outbreak in region P, 96.2% of total cases were from area A of region P, and in region X's outbreak, 88.1% of cases were found in a factory in area A of region X. In the outbreak in region Z, 97.4% of cases were in area A of region Z (Figure 2).

As for the population distribution, it shows that there are no significant differences among gender groups during the outbreaks in all four regions which was 58:71 (χ^2^ = 1.310, *p* = 0.252) in region H, 88:121 (χ^2^ = 3.211, *p* = 0.22) in region P, 116:120(χ^2^ = 0.068, *p* = 0.795) in region X, and 12:25 (χ^2^ = 3.789, *p* = 0.052) in region Z. But there are some differences among age groups. The median ± interquartile range (IQR) of regions H, P, X, and Z are 34 ± (15–48.5), 32 ± (9–46), 39 ± (31–47), and 21.5 ± (4–35), respectively. The differences in the age distribution of disease occurrence in the four regions were found to be statistically significant through rank-sum tests of multiple groups of samples (H = 40.9, *p* < 0.05). Vaccination also varied by region (χ^2^ = 28.907, *p* < 0.05). The proportions of the unvaccinated group were 46.5, 45.9, 12.7, and 13.2% in regions H, P, X, and Z, respectively. Disease severity varied in regions as well (χ^2^ = 10.907, *p* < 0.05). Nevertheless, it is worth noting that most cases reported in regions comprised of individuals with minor or ordinary symptoms, with fewer asymptomatic, heavy, or critical symptoms occurring (Table 2).

**Table 2:** Population distribution of COVID-19 outbreaks in four regions.

**Characteristics**		**Region H**		**Region P**		**Region X**		**Region Z**		**Statistical tests**

		***N***	**%**	***N***	**%**	***N***	**%**	***N***	**%**	
Total number of cases	129		471		236		38		
Gender	Male	58	45.0	88	42.1	116	49.2	13	34.2	(*χ^2^* = 2.480, *p* = 0.3501)
	Female	71	55.0	121	57.8	120	50.8	25	65.8	
Age	0–9	19	14.7	60	28.7	15	6.4	14	36.8	
	10–19	23	17.8	23	11.0	17	7.2	4	10.5	
	20–29	7	5.4	13	6.2	20	8.5	5	13.2	
	30–39	35	27.1	50	23.9	73	30.9	10	26.3	
	40–49	15	11.6	26	12.4	30	29.7	3	7.9	
	50–59	19	14.7	24	11.5	28	11.9	2	5.3	
	60–69	10	7.8	8	3.8	6	2.5	0	0.0	
	≥70	1	0.8	5	2.4	7	3.0	0	0.0	
Median age (IQR)		34 (15–48.5)		32 (9–46)		39 (31–47)		21.5 (4–35)		(H = 40.9, *p < * 0.05)
Vaccination	Unvaccinated	60	46.5	96	45.9	30	12.7	5	13.2	(*χ^2^* = 28.907, *p* < 0.05)
	1 Dose	23	17.8	10	4.8	11	4.7	4	10.5	
	2 Doses	45	34.9	103	49.3	195	82.6	17	44.7	
	3 Doses	1	0.8	0	0.0	0	0.0	12	31.6	
Disease severity	Asymptomatic	19	14.7	3	14	0	0	3	7.9	(*χ^2^* = 10.907, *p* < 0.05)
	Minor	31	24.0	84	40.2	50	21.2	26	68.4	
	Ordinary	78	60.5	116	55.5	176	74.6	9	23.7	
	Heavy	1	0.8	3	1.4	9	3.8	0	0	
	Severe	0	0	1	0.5	1	0.4	0	0	

### Calculating COVID-19 reproduction number

#### The definition method

**Figure 3:**
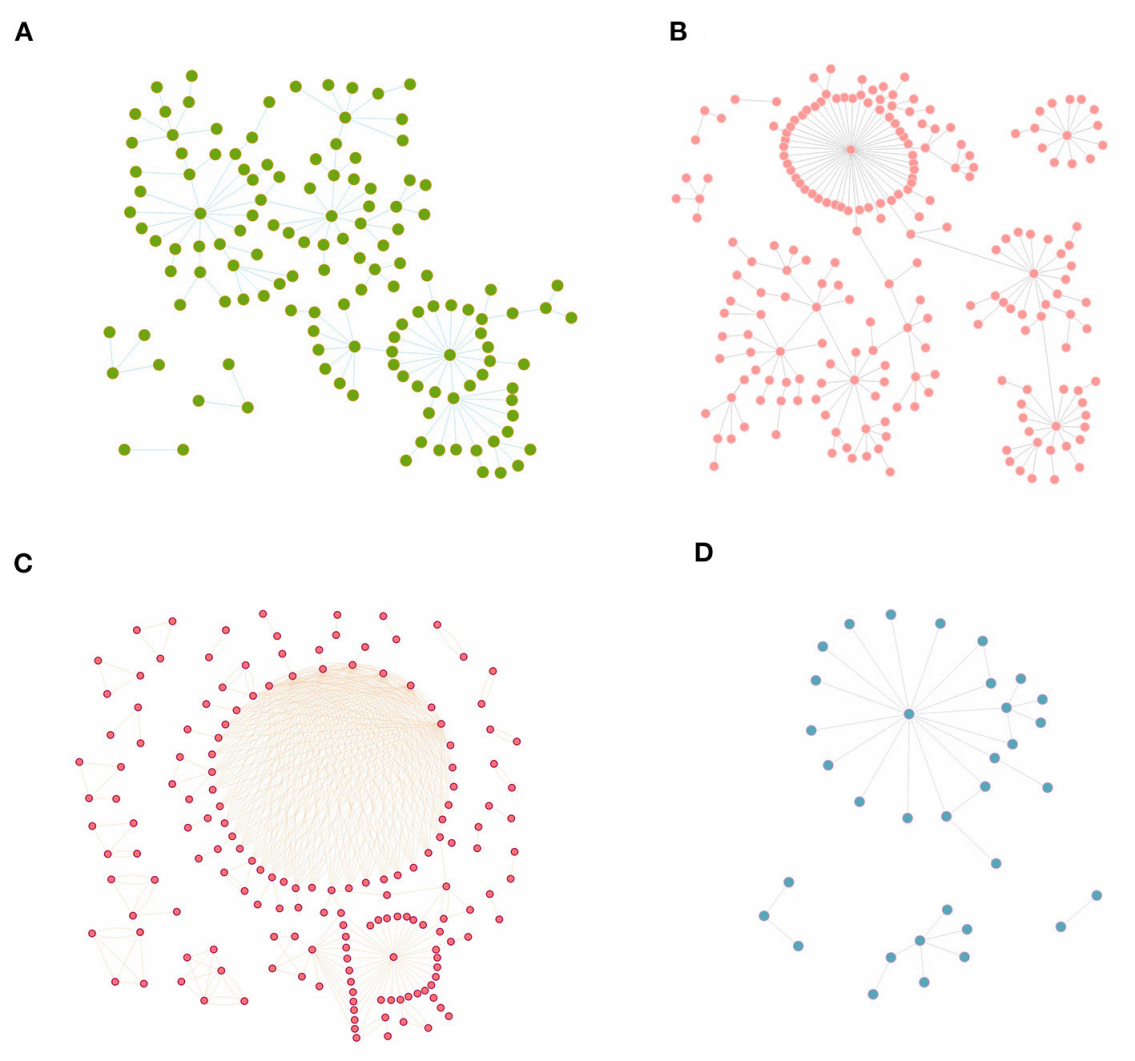
The transmission chains for COVID-19 outbreaks in four regions studied in this research. The dots in each figure represent the COVID-19 cases during each outbreak, while the bars between dots mean that these cases were on the same transmission chain. **(A)** Region H, **(B)** Region P, **(C)** Region X, and **(D)** Region Z.

**Figure 4:**
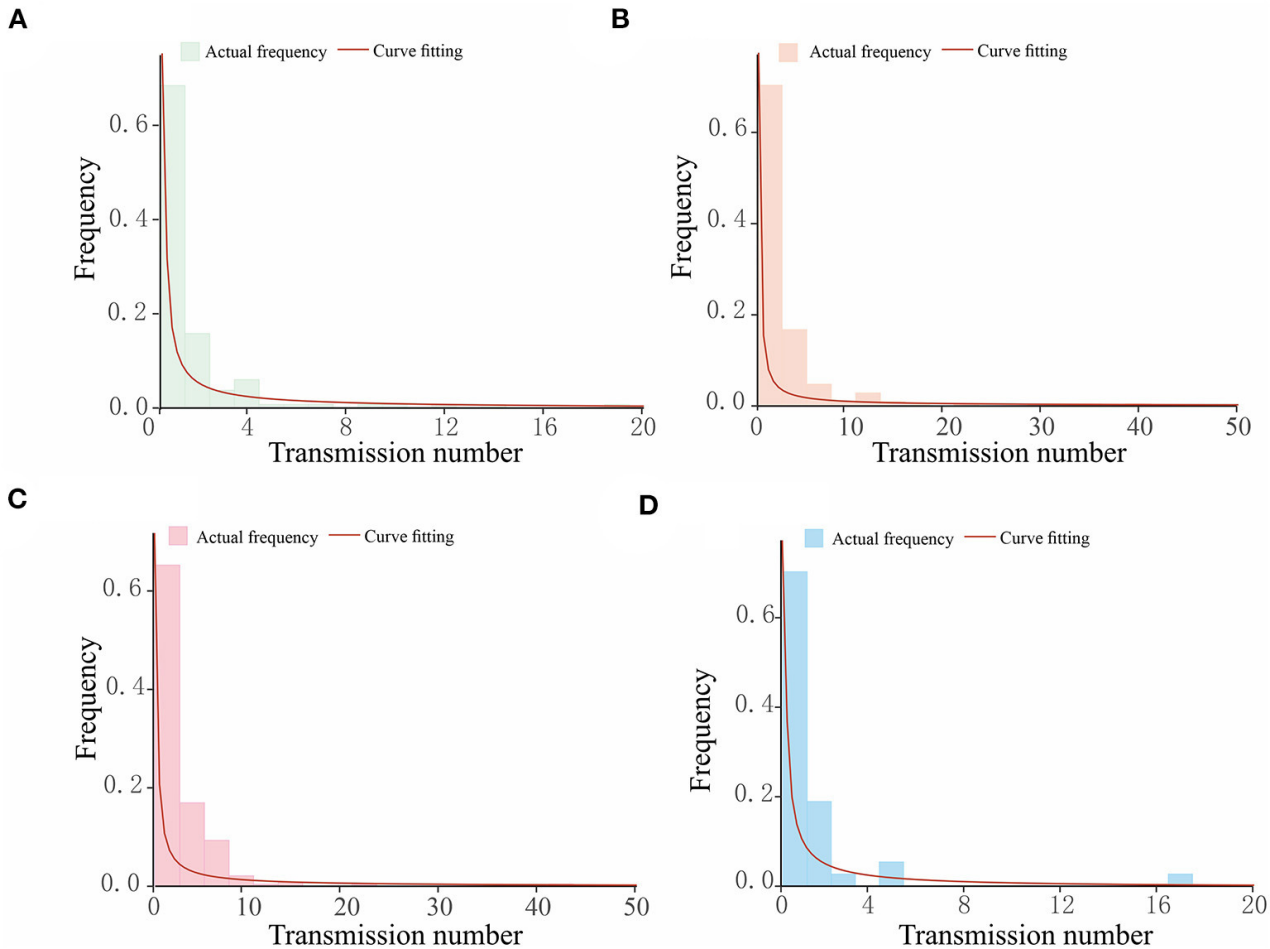
The gamma distribution for the transmission chains for COVID-19 outbreaks in four regions studied in this research. The horizontal axis in each figure shows the number of transmissions caused by a single case, while the vertical axis is the frequency of the transmission number. The bars present the actual frequency in each outbreak, and the red curves are the curves of fitting, which follow a gamma distribution. **(A)** Region H, **(B)** Region P, **(C)** Region X, and **(D)** Region Z.

After clarifying the transmission chains in the four outbreaks (Figure 3), we directly calculated the reproduction numbers of COVID-19 in those four regions. However, there would have been interventions implemented as soon as the authorities discovered a COVID-19 case, so the effective reproduction number *R*_*eff*_ has been used in this case rather than the basic reproduction number *R*_0_. Consequently, the effective reproduction number *R*_*eff*_ in regions H, P, X, and Z is *R*_*eff*_ = 1.20, *R*_*eff*_ = 1.14, *R*_*eff*_ = 1.66, and *R*_*eff*_ = 1.12, respectively. Notably, the reproduction numbers were far less than previous studies on the Delta and Omicron variants, which showed that the reproduction number is *R* = 5 for the Delta variant and *R* = 5–7 for the Omicron variant. Reproduction numbers of COVID-19 obtained from the definition method in the four regions were over 1, and although this means there were possibilities that the variants would cause outbreaks or epidemics, the speed of transmission was far lower than a super-spreading scenario. This is significant because the government and other departments would have taken prevention and control measures after detecting the cases for the first time, which ultimately presents a gamma distribution in the transmission chain (Figure 4).

#### The next-generation matrix-based method

**Figure 5:**
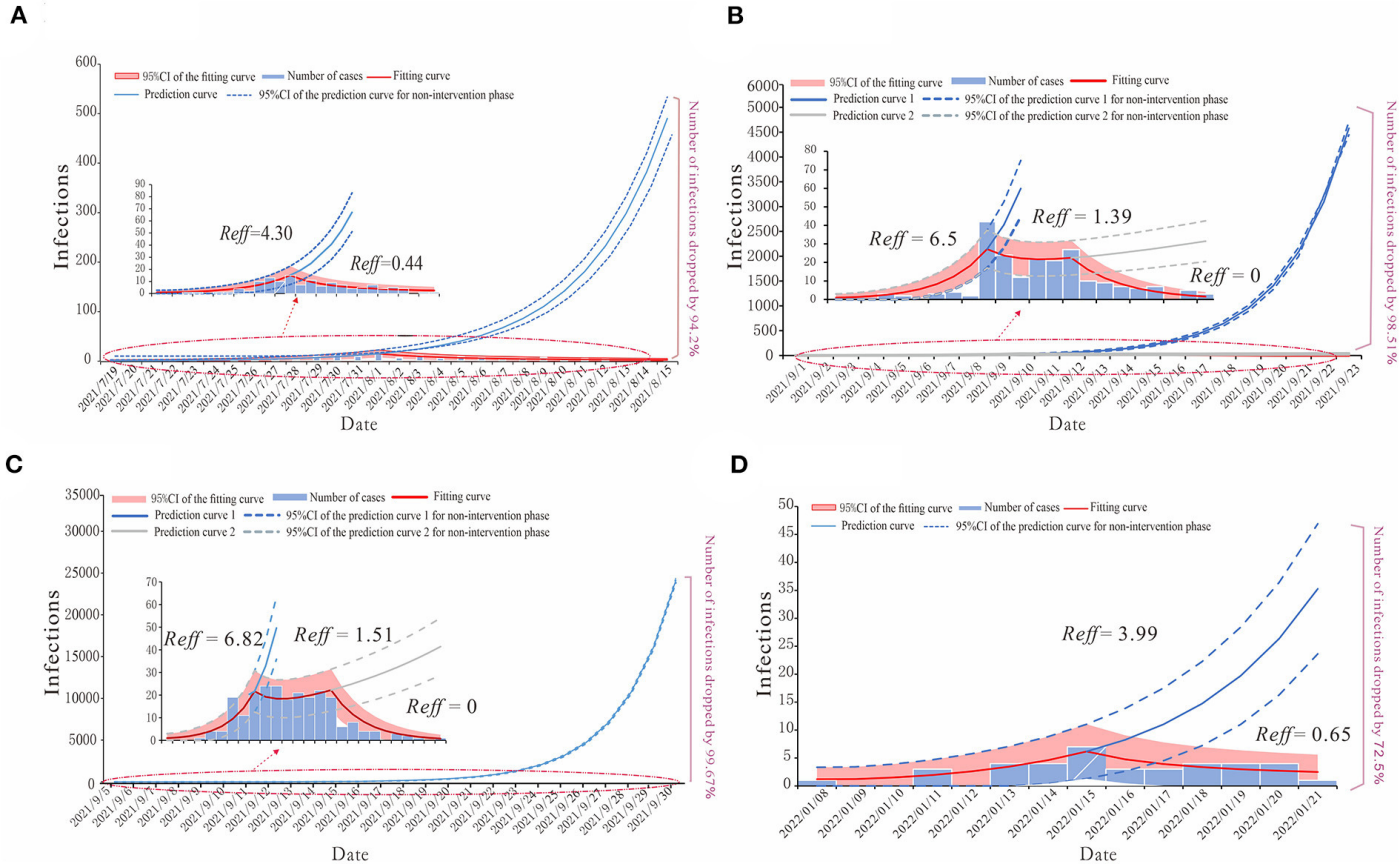
The simulation results of epidemic trends based on the different values of *R*_*eff*_ in each region. **(A)** Region H, **(B)** Region P, **(C)** Region X, and **(D)** Region Z.

We built a SEIAR transmission dynamics model and used the next-generation matrix to calculate the *R*_*eff*_ of COVID-19 in each of the four regions (Figure 5). The model fitted well for all four COVID-19 outbreaks in region H (*R*^2^ = 0.782, *p* < 0.001), P (*R*^2^ = 0.712, *p* < 0.001), X (*R*^2^ = 0.837, *p* < 0.001), and Z (*R*^2^ = 0.634, *p* < 0.001).

For region H, the COVID-19 outbreak by the Delta variant in July 2021 could be divided into the natural transmission stage (July 15–August 1) whose *R*_*eff*_ = 4.30 and the effective control stage (August 2–August 15) was *R*_*eff*_ = 0.44. We have predicted that without any proper interventions, there would have been a total of 2,226 cases (95% CI: 1,871–2,586), while in fact, region H reported 129 cases, which means that taking effective prevention and control measures helped decrease 94.2% of the potential infections (Figure 5A).

The epidemic curves of outbreaks in regions P and X, which were caused by the Delta variant, could be divided into three stages. The first stage is denoted as the natural transmission stage. In region P, it was from 4 to 10 September, with *R*_*eff*_ = 6.5; in region X, it was from 8 to 13 September, with *R*_*eff*_ = 6.82. The second stage is the effective containment stage. In region P, it was from 11 to 15 September, with *R*_*eff*_ = 1.39; in region X, it was from 14 to 20 September, with *R*_*eff*_ = 1.51. The last stage is the effective control stage. In region P, it was from 16 to 23 September; in region X, it was from 21 to 30 September, both with *R*_*eff*_ = 0. Our prediction illustrated the significance of effective intervention measures *via* different effective reproduction numbers of three stages in both regions P and X. In the case of region P, if the public health departments had failed to implement interventions by 10 September and the outbreak had continued to develop with *R*_*eff*_ = 6.5, there might have been 14,076 (95% CI: 13,345–14,809) cases by 23 September, or if they had not strengthened the intervention measures at the effective containment stage, there would have been 400 (95% CI: 236–588) cases by 23 September. The actual number of COVID-19 cases in region P was 208, thus, after strict interventions on the disease transmission, 98.51 and 49.02% of potential infections were prevented (Figure 5B). Similarly, in the case of region X, if the government had not taken any measures for prevention and control, there might have been 71,930 (95%CI: 70,301–73,562) cases by 30 September, with *R*_*eff*_ = 6.82 for the virus, or if they had not maintained the strict intervention measures, by 30 September, the total number of cases would have been 518 (95%CI: 308–730). However, in fact, there were 236 cases in this COVID-19 outbreak in region X, thus, the effective reproduction numbers show that the prevention and control measures prevented 99.67 and 54.44% of potential cases in the first and second stages of this outbreak, respectively (Figure 5C).

For the COVID-19 outbreak in region Z, which was caused by the Omicron variant, similar to that of region H, we have divided its epidemic curve into two stages, with the first stage being the natural transmission stage from 13 to 15 January, where *R*_*eff*_ = 3.99, and the second stage denoted as the effective control stage from 16 to 21 January, where *R*_*eff*_ = 0.65. If no cases had been detected in region Z before 15 January and no measures had been taken, there would have been a total of 138 (95% CI: 66–213) cases by 21 January. The actual number of cases reported was 38, which shows that the timely measures taken reduced potential cases by 72.5% (Figure 5D).

#### The epidemic curve and *SI*-based method

**Figure 6:**
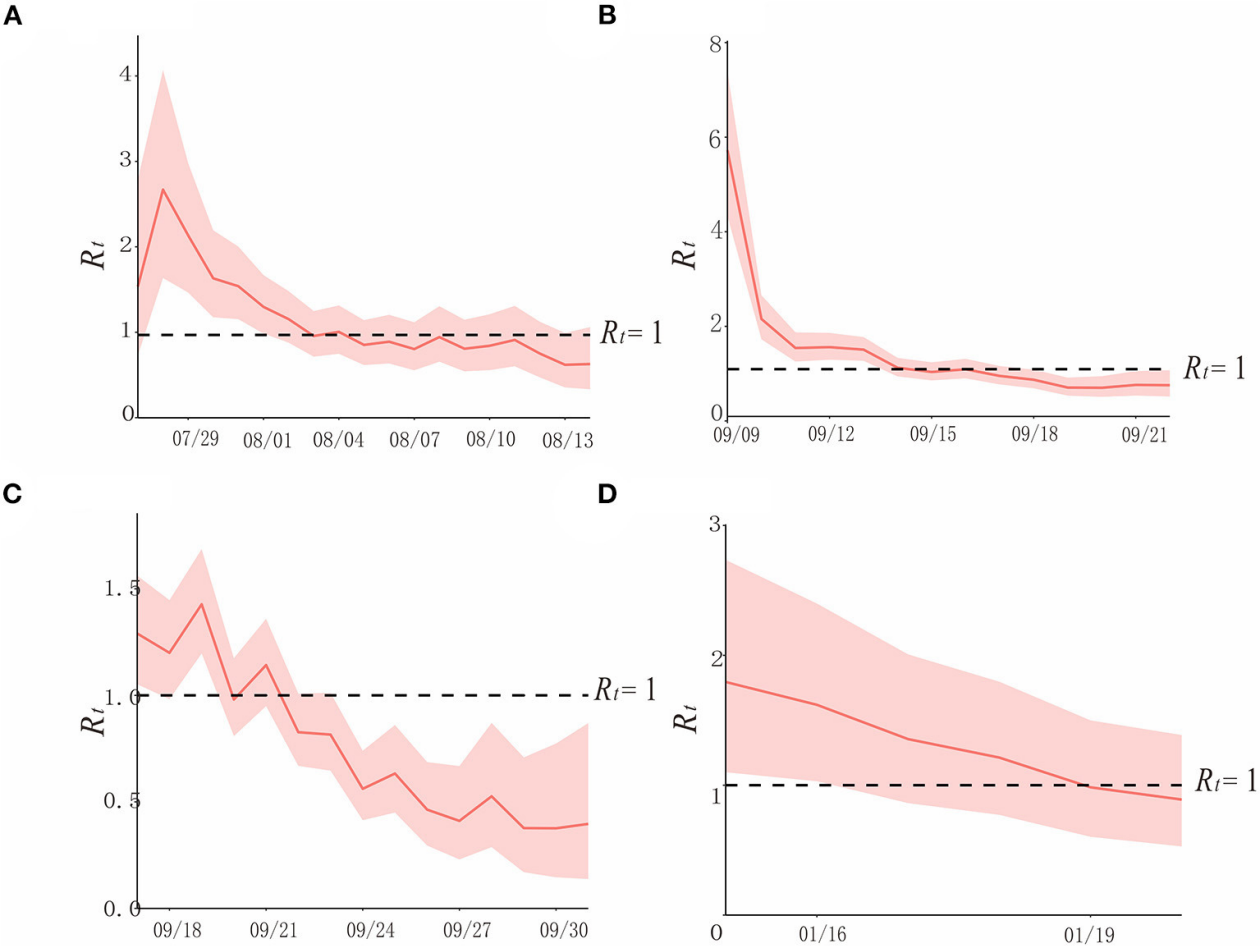
The simulation results of epidemic trends based on the different values of *R*_*t*_ in each region. **(A)** Region H, **(B)** Region P, **(C)** Region X, and **(D)** Region Z.

According to the statistical analysis, we have found that for the COVID-19 outbreaks by Delta variant in regions H, P, and X, the average *SI* of onset date is 2.3, and the standard deviation is 3.4. For the COVID-19 outbreak by the Omicron variant in region Z, the average *SI* and its standard deviation are 2.9 and 2.4, respectively. Then, with the combination of the epidemic curve and *SI*, we obtained *R*_*t*_ for the COVID-19 outbreaks in regions H, P, X, and Z (Figure 6).

In region H, at the beginning of the epidemic (before 29 July), when there were no interventions, and *R*_*t*_ varies from 1.5 to 3, showing a rapidly increasing trend. After 1 August, when prevention and control measures were taken, *R*_*t*_was declining, it was below 1 after 3 August, and continued until the end of the outbreak. Though the curve approached *R*_*t*_ = 1 on 8 and 11 August, it did not exceed it, due to the strict policies in place (Figure 6A).

In region P, according to the curve, *R*_*t*_ was between 5 and 6 at the beginning of the epidemic. Interventions had been implemented after detecting the first case on 10 September, which contributed to the rapid decline of the transmissibility of the variant, and it is consistent with the effective containment stage in the epidemic curve. After 14 September, *R*_*t*_ < 1, and it lasted until the end of this outbreak (Figure 6B).

In region X, which experienced the first factory cluster outbreak by the Delta variant of COVID-19 in China, the trend of its *R*_*t*_ curve fluctuates, yet it did not exceed 1.5. *R*_*t*_and appeared to be declining after the public health department took prevention and control measures. *R*_*t*_ was <1 after 23 September, and it was consistent with the effective control stage (Figure 6C).

*R*_*t*_ of COVID-19 caused by the Omicron variant in January 2022 in region Z was over 1.5 before the administrations took effective measures on 15 January, which is a sign of a potential epidemic. After strict interventions, *R*_*t*_ decreased gradually, and it was below 1 on 19 January, and this outbreak was contained (Figure 6D).

## Discussion

Evaluating the transmissibility of COVID-19 is of significance in the prevention and control of the pandemic. In this study, we obtained the reproduction numbers of COVID-19 through the definition method, the next-generation matrix-based method, and the *SI* and epidemic curve-based method, with outbreak data collected from four different regions of Delta and Omicron variants of SARS-CoV-2. Then, we made a comparison of the reproduction numbers obtained from different methods and looked into the merits and demerits of those calculation methods and their reproduction numbers in actual situations, so as to provide public health departments with a valid and reliable index for evaluating the transmissibility of COVID-19 and a theoretical basis for implementing intervention measures.

In selecting the outbreak data, we have chosen the outbreaks in region H in July 2022, in region P and X in September 2021, and in region Z in January 2022. For the past 2 years, SARS-CoV-2 has undergone mutations, where transmissibility varies with the various strains. Before November 2021, most of the outbreaks in China were caused by the Delta variant. After this, it was the Omicron variant that became the dominant variant worldwide (2, 26). In this study, outbreaks in regions H, P, and X were caused by the Delta variant, while the outbreak in region Z was caused by the Omicron variant. On the scale of the epidemic, outbreaks in regions H and P were observed to have a similar scale as the previous epidemics in China. As for the outbreak in region X in September 2021, this was the first factory cluster outbreak caused by the Delta variant in China. However, as for the outbreak in region Z in January 2022, though it was on a small scale, it is informative in relation to small outbreaks that may occur.

For the time distribution, it is concluded that outbreaks in all four regions showed a multi-phased characteristic, which is similar to most of the epidemics previously. As for the population and spatial distribution of the outbreaks in the four regions, it is confirmed that the population is susceptible to all COVID-19 variants, with no differences between Delta and Omicron variants. There was no difference in gender, although the age of the infected patients was mainly in the range of 20–39 years old. As for the disease severity, it is noteworthy that during the outbreaks caused by the Delta variant, there were cases of heavy symptoms, while in the outbreak of the Omicron variant, there were no heavy symptoms or serious cases; however, this may not be significant considering that there was only one data set of the Omicron variant in this study.

By obtaining the reproduction numbers through three different approaches, namely, the definition method, the next generation matrix-based method, and the *SI* and epidemic curve-based method, which is the main purpose of this study, we made comparisons between the different reproduction numbers (Table 3).

**Table 3:** Reproduction numbers in four regions.

**Various phases of outbreaks**		**The definition method (*R_*eff*_*)**	**Method based on next-generation matrix (*R_*eff*_*)**	**Method based on epidemic curve and serial interval (*R_*t*_*)**
Region H	Phase 1 (d1 ~ d10)	1.20	4.30	1.5–3
	Phase 2 (d11 ~ d26)		0.44	< 1
Region P	Phase 1 (d1 ~ d6)	1.14	6.51	5–6
	Phase 2 (d7 ~ d11)		1.39	1–5
	Phase 3 (d12 ~ d20)		0	< 1
Region X	Phase 1 (d1 ~ d5)	1.66	6.82	>1.5
	Phase 2 (d6 ~ d12)		1.51	< 1.5
	Phase 3 (d13 ~ d24)		0	< 1
Region Z	Phase 1 (d1 ~ d3)	1.12	3.99	>1.5
	Phase 2 (d4 ~ d9)		0.65	0.8–1.8

For the effective reproduction number, *R*_*eff*_, obtained from the definition method, it is calculated that *R*_*eff*_ = 1.20, 1.14, 1.66, and 1.12 in regions H, P, X, and Z, respectively. The first three *R*_*eff*_s were caused by the Delta variant, and *via* the *R*_*eff*_, which is relatively low, it showed that one infection could infect at least one susceptible individual. In previous studies, it was indicated that *R*_0_ for the Delta variant was usually 5, and for the Omicron variant it was 5–7 (22, 27), which is significantly different from that in this study. Although we obtained *R*_*eff*_ that is consistent with the condition of transmission of the virus through the definition method, it is much slower than the real scenario. According to our analysis, it was found that the transmission chain in the cases was incomplete; in addition, as soon as the first case was reported, the government would have taken strict interventions against the epidemic, which may have resulted in bias in the results. Thus, it is concluded that the reproduction number obtained through the definition method cannot perform well in illustrating the transmissibility of the disease after interventions have been implemented; that is, it is considered that the definition method for calculating the reproduction number should be conducted in the early stages of the epidemic when there are clear transmission chains to predict the trend of the infectious disease (28).

Driven by the Chinese policy of preventing and controlling the transmission of SARS-CoV-2, appropriate interventions would have been implemented by the local government soon as a case was reported. Therefore, calculating the reproduction number, which is denoted as *R*_*eff*_, through the next generation matrix method by using the transmission dynamics models is effective, because it can provide us with references for the trend of the epidemic and evaluate the effects of the conducted measures (8). The reproduction numbers in the outbreak of region P are taken as an example. According to the fitting curve, the outbreak could be divided into three phases. The first phase was the natural transmission phase, where *R*_*eff*_ = 6.5, and it explains the rapid increase of cases in the early stage of the outbreaks. When relevant interventions were taken, this was the second phase, where *R*_*eff*_ = 1.39, and although it shows that the transmissibility of the virus is slower than phase I, the virus was still able to transmit in region P and the outbreak continued. With the implementation of intervention measures were strengthen, this was the third phase, where *R*_*eff*_ = 0, and it showed that the outbreak was under control and there was no way that the disease would still spread in the city. In conclusion, it was found that the reproduction number from the next-generation matrix method was able to forecast and simulate the trend of the epidemic to evaluate the prevention and control measures more validly and offer significant references for future epidemics.

The time-varying reproduction number, obtained *via* epidemic curve and *SI*, presents the dynamic change of the epidemic and is now used more frequently (27, 29). However, the condition required for calculating *R*_*t*_ is more complicated: we have to recognize a clear transmission chain and obtain the *SI* between the first-generation cases and second-generation cases. In this study, we found that the SI of onset date for the Delta variant of the outbreaks in regions H, P, and X was 2.4 ± 3.4, which is smaller than that in Guangdong Province. The reason for this bias may be due to the lack of transmission chain in this study. While for the Omicron variant, whose onset date *SI* in region Z is 2.9 ± 2.4, in this outbreak, the transmissibility of the variant is less strong. However, because the total number of cases in region Z was much smaller, it is unrealistic to generalize the result in this case. As *R*_*t*_ is a real-time index, it is more instructive to use it as a reference when assessing the trend of epidemics to focus on prevention and controls. However, lacking transmission chains or losing the partial case data would lead to biases in calculating *R*_*t*_, and, worse, could mislead decision-makers in connection to implementing intervention measures.

**Figure 7:**
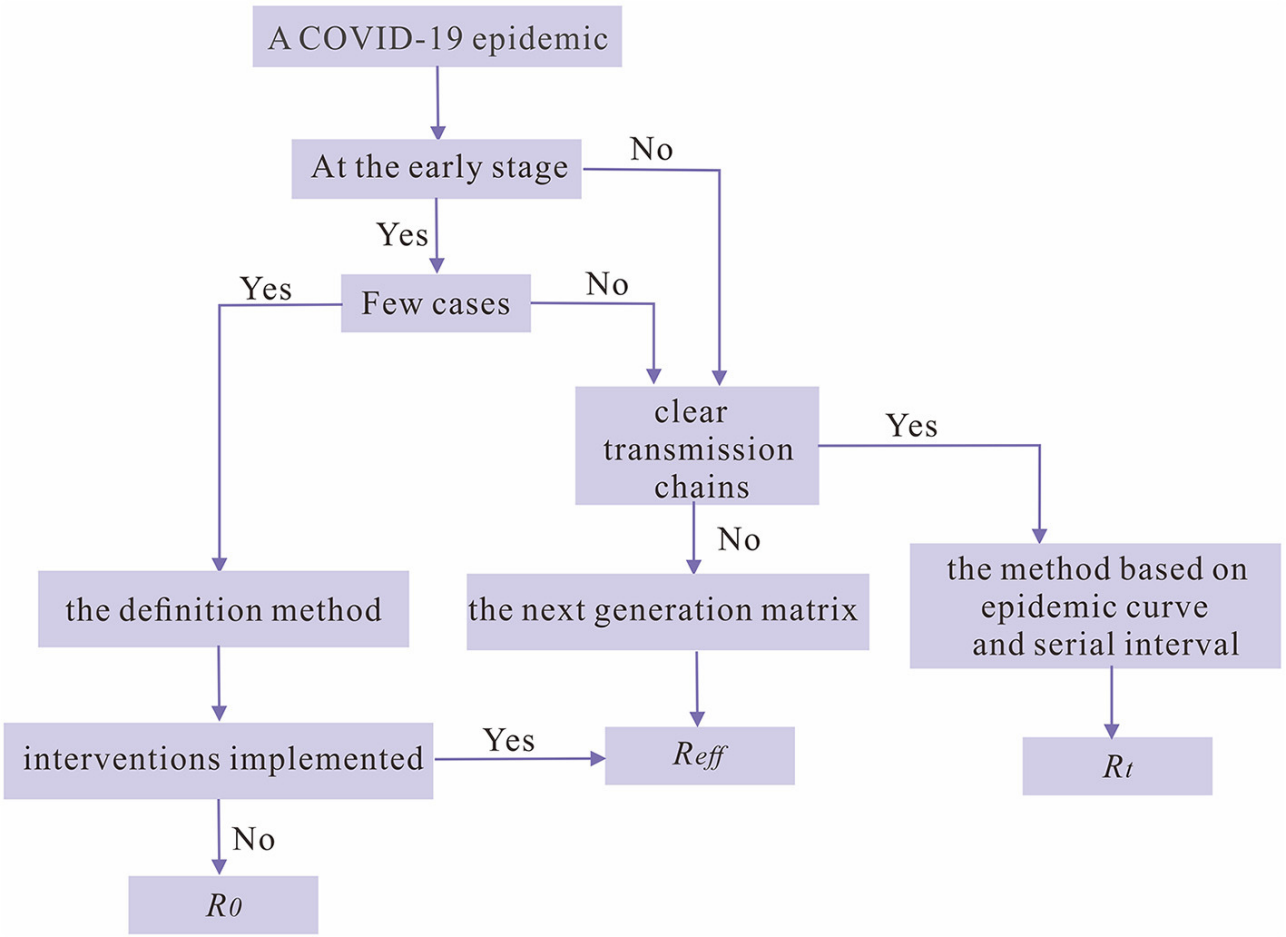
The decision tree for choosing the optimal reproduction number as an index for the transmissibility of SARS-CoV-2 during a COVID-19 epidemic.

According to the above discussion, we have summarized with a decision tree to assist researchers to choose an optimal method for calculating reproduction numbers when there is a COVID-19 epidemic and to provide references for intervention implementation (Figure 7).

## Limitations

Due to technical conditions and data limitations, this study has the following limitations. First, the data used in this study are one provincial Delta variant outbreak, two municipal Delta variant outbreaks, and one municipal Omicron variant outbreak. As a comparative study, data information on the corresponding scale of the two types of variants should be guaranteed. Second, there are several ways to calculate the reproduction number, but in this study, only three methods were used for the calculation of the reproduction number, which can be further calculated by different methods and further compared to draw more reliable conclusions.

## Conclusion

In this study, we calculated the reproduction numbers of four Chinese COVID-19 outbreaks by using the definition method, the next-generation matrix-based method, and the epidemic curve and *SI*-based method, then analyzed and compared the best applicable scenarios for each of these methods. The definition method is generally used in the early stages of an epidemic, when the number of cases is small, to be able to assess the virus transmissibility and the effectiveness of initial prevention and control measures and to be able to predict the trend of the epidemic, but the results may be biased when the transmission chain is unclear and the number of cases is too large. The method based on the next-generation matrix is mostly used in situations where there are more cases, and the transmission chains are unclear. The index incorporates a variety of factors to control transmission, which can well evaluate the measures taken for epidemic prevention and control, to make a judgment on the scale of the epidemic, but for larger-scale epidemic outbreaks, this method has some limitations. For the calculation of the reproduction number obtained using the method based on the epidemic curve and *SI, R*_*t*_ can give the real-time spread of COVID-19, making it easier and more efficient for decision-makers to take intervention measures, but the method is more complex and requires harsh conditions, and it needs enough clear generational relationships to calculate the *SI* so that the reproduction number can be calculated. Through the rational use of different reproduction number methods, the benefits of prevention and control measures can be maximized, and a good basis for further clarifying the epidemiological significance of disease transmission can be created.

## Data availability statement

The original contributions presented in the study are included in the article/Supplementary material, further inquiries can be directed to the corresponding authors.

## Author contributions

TC, BA, WL, ZG, and CS designed research. BA, WL, and ZZ analyzed data. TC, CS, ZG, WL, BA, ZZ, JR, WS, YW, QC, and RF conducted the research and analyzed the results. TC, CS, WL, ZG, and BA wrote the manuscript. All authors read and approved the final manuscript.
