# Supplementary material for: A comparative study on the three calculation methods for reproduction numbers of COVID-19
Source: Front Med (Lausanne). 2023 Jan 5;9:1079842. doi: 10.3389/fmed.2022.1079842 (PMC9849755; doi:10.3389/fmed.2022.1079842)
Supplement: Supplementary file 1 [file Data_Sheet_1.docx]

Supplementary Material

1. **Detailed calculation for *R_eff_* through the transmission dynamics model**

SEIAR (Susceptible- Exposed- Infections- Asymptomatic - Recovered/Removed) model

1. Flowchart


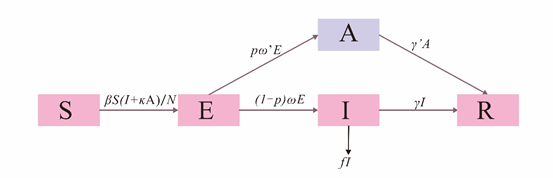


1. Equations

$$\begin{matrix} & \frac{dS}{dt}=-\beta S\left( I+\kappa A \right)/N \\ & \frac{dE}{dt}=\beta S\left( I+\kappa A \right)/N-\left( 1-p \right)\omega E-p\omega^{'}E \\ & \frac{dI}{dt}=\left( 1-p \right)\omega E-\gamma I-fI \\ & \frac{dA}{dt}=p\omega^{'}E-\gamma^{'}A \\ & \frac{dR}{dt}=\gamma I+\gamma^{'}A \end{matrix}$$

2. Calculation for the reproduction number
3. Definition-Based Method for SEIAR

(1) For any infected individual $x$, it is initially developed into $E$. Thus, we would discuss from $E$. The probabilities that $x$ attains each infectious compartment are:

$$\begin{matrix} \mathbb{P}\left( x\in A \right)= & \mathbb{P}\left( x\in A|x\in E \right)\mathbb{P}\left( x\in E \right)=\frac{p\omega^{'}}{p\omega^{'}+\left( 1-p \right)\omega} \\ \mathbb{P}\left( x\in I \right)= & \mathbb{P}\left( x\in I|x\in E \right)\mathbb{P}\left( x\in E \right)=\frac{\left( 1-p \right)\omega}{p\omega^{'}+\left( 1-p \right)\omega} \end{matrix}$$

where $\mathbb{P}\left( x\in I|x\in E \right)$ denote the conditional probability of $x\in I$ with $x\in E$ known.
(2) Letting one of components of $\left( I_{i},A_{i} \right)$ equals to 1 and others equal to 0, and substitute in the newly infection term $\beta S\left( I+\kappa A \right)/N$, one obtains the secondary infection that $x$ will produces in unit time in different states:

$$Q\left( x \right)=\left\{ \begin{matrix} \kappa\beta S/N, & \text{ if }x\in A \\ \beta S/N, & \text{ if }x\in I \end{matrix} \right.$$

(3) The infectious period is given by:

$$T\left( x \right)=\left\{ \begin{matrix} 1/\left( \gamma^{'} \right), & \text{ if }x\in A \\ 1/\left( f+\gamma\right), & \text{ if }x\in I \end{matrix} \right.$$

(4) At the beginning of disease, $S\approx N$. As a good approximation, it is natural to assume tha $Q\left( x \right)$ remain constant during time interval $T\left( x \right)$. Therefore, the secondary infections that $x$ will produce during its lifespan as infectious is given by:

$$\begin{matrix} R_{eff}= & \mathbb{P}\left( x\in A \right)\times Q\left( x\in A \right)\times T\left( x\in A \right)\mathbb{+P}\left( x\in I \right)\times Q\left( x\in I \right)\times T\left( x\in I \right) \\ = & \frac{\beta S/N}{p\omega^{'}+\left( 1-p \right)\omega}\left[ \frac{\kappa p\omega^{'}}{\gamma^{'}}+\frac{\left( 1-p \right)\omega}{\gamma+f} \right] \end{matrix}$$

(5) Assuming all individuals are susceptible, and substituting $S=N$, one obtains:

$$R_{0}=\frac{\beta}{p\omega^{'}+\left( 1-p \right)\omega}\left[ \frac{\kappa p\omega^{'}}{\gamma^{'}}+\frac{\left( 1-p \right)\omega}{\gamma+f} \right]$$

1. Next generation method for SEIAR

(1) Divide (S,E,I,A,R) into two categories: the first (E,I,A) are infected, and the second (S,R) are uninfected.
(2) Divide the derivatives of (E,I,A) into two parts: the first $\mathcal{F}$ represents rates of newly infections, and the second $\mathcal{V}$ represents rates of transition between (E,I,A):

$$\begin{matrix} \frac{d}{dt}\left[ \begin{matrix} E \\ I \\ A \end{matrix} \right]=\left[ \begin{matrix} \beta S\left( I+\kappa A \right)/N-p\omega^{'}E-\left( 1-p \right)\omega E \\ \left( 1-p \right)\omega E-\left( f+\gamma\right)I \\ p\omega^{'}E-\gamma^{'}A \end{matrix} \right] & =\left[ \begin{matrix} \beta S\left( I+\kappa A \right)/N \\ 0 \\ 0 \end{matrix} \right]-\left[ \begin{matrix} p\omega^{'}E+\left( 1-p \right)\omega E \\ -\left( 1-p \right)\omega E+\left( f+\gamma\right)I \\ -p\omega^{'}E+\gamma^{'}A \end{matrix} \right] \\ & :=\mathcal{F-V} \end{matrix}$$

(3) Take derivatives of the vector-valued function $\mathcal{F}$ and $\mathcal{V}$ respect to $\left( E,I,A \right)$, the Jacobi matrices are obtained:

$$F=\left( \begin{matrix} 0 & \kappa\beta S/N & \beta S/N \\ 0 & 0 & 0 \\ 0 & 0 & 0 \end{matrix} \right)$$

$$\begin{matrix} V=\left( \begin{matrix} \omega^{'} p-\omega\left( p-1 \right) & 0 & 0 \\ -\omega^{'} p & \gamma^{'} & 0 \\ \omega\left( p-1 \right) & 0 & f+\gamma\end{matrix} \right) \end{matrix}$$

The inverse of V is further computed:

$$V^{-1}=\left( \begin{matrix} \frac{1}{-\omega p+\omega+\omega^{'} p} & 0 & 0 \\ \frac{\omega^{'} p}{\gamma^{'} \left( -\omega p+\omega+\omega^{'} p \right)} & \frac{1}{\gamma^{'}} & 0 \\ -\frac{\omega\left( p-1 \right)}{\left( f+\gamma\right) \left( -\omega p+\omega+\omega^{'} p \right)} & 0 & \frac{1}{f+\gamma} \end{matrix} \right)$$

(4) Construct the next generation matrix $M=FV^{-1}$:

$$M=FV^{-1}=\left( \begin{matrix} \frac{S \beta\kappa\omega^{'} p}{N \gamma^{'} \left( -\omega p+\omega+\omega^{'} p \right)}-\frac{S \beta\omega\left( p-1 \right)}{N \left( f+\gamma\right) \left( -\omega p+\omega+\omega^{'} p \right)} & \frac{S \beta\kappa}{N \gamma^{'}} & \frac{S \beta}{N \left( f+\gamma\right)} \\ 0 & 0 & 0 \\ 0 & 0 & 0 \end{matrix} \right)$$

whose leading eigenvalue:

$$R_{eff}=\lambda_{max}\left( FV^{-1} \right)=\frac{S \beta\left( f \kappa\omega^{'} p+\gamma\kappa\omega^{'} p-\gamma^{'} \omega p+\gamma^{'} \omega\right)}{N \gamma^{'} \left( f+\gamma\right) \left( -\omega p+\omega+\omega^{'} p \right)}$$

(5) Substitute the disease-free equilibrium (S,E,A,I,R) = (N,0,0,0,0), one obtains:

$$R_{0}=\frac{\beta\left( f \kappa\omega^{'} p+\gamma\kappa\omega^{'} p-\gamma^{'} \omega p+\gamma^{'} \omega\right)}{\gamma^{'} \left( f+\gamma\right) \left( -\omega p+\omega+\omega^{'} p \right)}$$

by few steps of simplification, one can see that the $R_{eff}$, $R_{0}$ derived by Next Generation Method is identical to the result of Definition Based Method.

That is:

$$\begin{matrix} & \\ R_{eff}= & \frac{\beta S/N}{p\omega^{'}+\left( 1-p \right)\omega}\left[ \frac{\kappa p\omega^{'}}{\gamma^{'}}+\frac{\left( 1-p \right)\omega}{\gamma+f} \right] \end{matrix}$$

$$R_{0}=\frac{\beta}{p\omega^{'}+\left( 1-p \right)\omega}\left[ \frac{\kappa p\omega^{'}}{\gamma^{'}}+\frac{\left( 1-p \right)\omega}{\gamma+f} \right]$$

1. Code to obtain the *R_eff_* value (Berkeley Madonna)
2. For Region H

METHOD RK4

STARTTIME = 0

STOPTIME=27

DT =0.02

d/dt (S)=-b1*S1*(I1+k1*A1);

d/dt (E)=b1*S1*(I1+k1*A1) -(1-p)*w1*E-p*w2*E;

d/dt (A)=p*w2*E1-y2*A;

d/dt (I)=(1-p)*w1*E-y1*I;

d/dt (R)=y1*I+y2*A;

N= S+E+I+A+R;

x=(1-p)*w1*E;

init S=66200000;

init E=3;

init I=1;

init A=0;

init R=0;

b=if time<=13 then b1 else b2;

b2=if time<=14 then b3 else b4;

b1=2.7872e-7;

b3=0.812105;

b4=0.083561;

p1=0.15;

w1=0.333;

w2=0.200;

y1=0.200;

y2=0.100;

1. For region P

METHOD RK4

STARTTIME = 0

STOPTIME=22

DT =0.02

d/dt (S)=-b1*S1*(I1+k1*A1);

d/dt (E)=b1*S1*(I1+k1*A1) -(1-p)*w1*E-p*w2*E;

d/dt (A)=p*w2*E1-y2*A;

d/dt (I)=(1-p)*w1*E-y1*I;

d/dt (R)=y1*I+y2*A;

N= S+E+I+A+R;

x=(1-p)*w1*E;

init S=3220000;

init E=3;

init I=1;

init A=0;

init R=0;

b=if time<=9 then b1 else b2;

b2=if time<=14 then b3 else b4;

b1=2.7872e-7;

b3=1.300001;

b4=0.278466;

p1=0;

w1=0.333;

w2=0;

y1=0.200;

y2=0;

1. Region X

METHOD RK4

STARTTIME = 0

STOPTIME=28

DT =0.02

d/dt (S)=-b1*S1*(I1+k1*A1);

d/dt (E)=b1*S1*(I1+k1*A1) -(1-p)*w1*E-p*w2*E;

d/dt (A)=p*w2*E1-y2*A;

d/dt (I)=(1-p)*w1*E-y1*I;

d/dt (R)=y1*I+y2*A;

N= S+E+I+A+R;

x=(1-p)*w1*E;

init S=5280000;

init E=3;

init I=1;

init A=0;

init R=0;

b=if time<=8 then b1 else b2;

b2=if time<=15 then b3 else b4;

b1=2.7872e-7;

b3=4.37575e-8;

b4=1.43036e-15;

p1=0;

w1=0.333;

w2=0;

y1=0.200;

y2=0;

1. Region Z

METHOD RK4

STARTTIME = 0

STOPTIME=13

DT =0.02

d/dt (S)=-b1*S1*(I1+k1*A1);

d/dt (E)=b1*S1*(I1+k1*A1) -(1-p)*w1*E-p*w2*E;

d/dt (A)=p*w2*E1-y2*A;

d/dt (I)=(1-p)*w1*E-y1*I;

d/dt (R)=y1*I+y2*A;

N= S+E+I+A+R;

x=(1-p)*w1*E;

init S=2440000;

init E=3;

init I=1;

init A=0;

init R=0;

b=0.885645;

p1=0;

w1=0.4;

w2=0;

y1=0.222;

y2=0;

1. **Detailed calculation for *R_t_* through the method based on the serial interval and epidemic curve**

Code to obtain the *R_t_* value (R)

# packages ----------------------------------------------------------------

library(EpiEstim)

library(tidyverse)

library(openxlsx)

# setting -----------------------------------------------------------------

mean_si <- N

std_si <- N

start_date <- N

space_date <- N

# prepare -----------------------------------------------------------------

datafile <- read.xlsx('./rt. xlsx', colNames = F)

names(datafile) <- c('dates', 'I')

datafile$dates <- convertToDate(datafile$dates)

date_st <- min(datafile$dates)

start_dates <- seq(start_date, nrow(datafile) - space_date)

end_dates <- start_dates + space_date

config_lit <- make_config(list(

mean_si = mean_si,

std_si = std_si,

t_start = start_dates,

t_end = end_dates

))

# estimate ----------------------------------------------------------------

epiestim_res_lit <- estimate_R(incid = datafile,

method = "parametric_si",

config = config_lit)

outcome <- epiestim_res_lit$R

outcome$date <- (outcome$t_start + outcome$t_end) / 2 + date_st

outcome$t_start <- date_st + outcome$t_start

outcome$t_end <- date_st + outcome$t_end

write.csv(outcome, file = "./rt.csv")
